# Glycemic control and its associated factors in type 2 diabetes patients at Felege Hiwot and Debre Markos Referral Hospitals

**DOI:** 10.1038/s41598-022-13673-5

**Published:** 2022-06-08

**Authors:** Nigusie Gashaye Shita, Ashagrie Sharew Iyasu

**Affiliations:** Department of Statistics, Debre Markos University, Debre Markos, Ethiopia

**Keywords:** Scientific data, Statistics, Risk factors

## Abstract

Poor glycemic control is a main public health problem among type 2 diabetes mellitus (T2DM) patients and a significant cause of the development of diabetic complications. This study aimed to assess the glycemic control status and its associated factors among type 2 diabetes patients in Felege-Hiwot and Debre Markos Referral Hospitals. A retrospective cohort study was conducted at Felege-Hiwot and Debre Markos Referral Hospitals from December 2014 to December 2015. We have reviewed the chart of these patients until January 2020. Type 2 diabetic patients on follow-up at Felege-Hiwot and Debre Markos Referral Hospitals who fulfilled the inclusion criteria of the study were included. The primary outcome was the level of blood glucose during the study period. *Good glycemic control* was defined as patients whose average fasting blood glucose measurement for three consecutive visits was between 70 and 130 mg/dL. A generalized linear mixed autoregressive order one model was used to identify the determinants of glycemic control. A total of 191 patients with 1740 observations were included in the study. The overall prevalence of good glycemic control was 58.4% (95% CI: 57.159.7%). The factors associated with good glycemic control at 95% confidence level adjusted odds ratio were being residing in rural **(**CI: 0.454, 0.614), negative proteinuria (CI: 1.211, 1.546), diastolic blood pressure < 90 (CI: 1.101, 1.522), systolic blood pressure < 140 (CI: 1.352, 1.895), serum creatinine (CI: 0.415, 0.660), duration per visit (CI: 0.913, 0.987), duration since diagnosis (CI: 0.985, 0.998), weight ≥ 78 kg (CI: 0.603, 0.881). Age 38–50, 51–59 and 60–66 years (CI: 1.267, 1.776), (CI: 1.057, 1.476) and (CI: 1.004, 1.403), respectively. The overall prevalence of poor glycemic control was high at Debre Markos and Felege Hiwot Referral Hospital. Living in a rural area, older age (≥ 67 years), positive proteinuria, higher weight (≥ 78 kg), higher serum creatinine levels, higher duration per visit, higher time duration of T2DM since diagnosis, and developing hypertension (SBP ≥ 140, DBP ≥ 90) were the predictors of lower good glycemic control achievements of T2DM patients. In response to this finding, an aggressive intervention that targets improving glycemic control is required.

## Introduction

Type 2 diabetes mellitus (T2DM) is a chronic disease. It is defined as high sugar in the blood and results from a defect in insulin secretion, insulin action, or both^1,2^.

Globally, the prevalence of diabetes was estimated to be 8.3% (6.2–11.8) in 2019 among individuals aged 20–79, including 50.1% who are undiagnosed. It will be 10.2% (8.1–13.2) by 2030. In African Region, with 59.7%, undiagnosed diabetes has a prevalence of 3.9% (2.1–7.1%) among individuals aged 20–79; it will be 4.1% (2.3–7.5%) by 2030. In Ethiopia, the prevalence of diabetes was predicted to be 3.2% of individuals aged 20–79 years in 2019^1,3^. Global burden of disease data suggests diabetes mellitus (DM) may be responsible for 4.2 million individuals aged 20–79 years death in 2019^1^.

T2DM is a rapidly rising non-communicable disease and a public health challenge in Ethiopia with disability and premature death due to the long-term effects of untreated diabetes mellitus^1,3^. Hence, glycemic control is a means of effectively preventing complications associated with T2DM^4^. However, the proportion of uncontrolled levels of blood sugar in T2DM was far above the ground in Ethiopia^5–9^.

The predictors of poor glycemic control were rural residence, age of the patients, duration with diabetes, time duration since diagnosis, drug regimen of oral anti-diabetics or insulin treatment, and body weight^5–8^. Yet, there is limited evidence on how these factors are associated with glycemic control using longitudinal data (repeated measured data) even if the glycemic control levels of T2DM patients fluctuate over time. Additionally, previous studies did not assess the influence of proteinuria and creatinine on glycemic control^5–8^.

Even if a few longitudinal studies were conducted on the predictors of blood glucose levels using fasting blood glucose levels as a continuous variable^9–11^, the findings of prior studies have not clear recommendations and conclusions about the controlled level of blood sugar of the patients because the negative predictors of fasting blood glucose levels led to hypoglycemia whereas the positive predictors of fasting blood glucose levels also lead to hyperglycemia.

Hence, this study aimed to investigate the glycemic control status and its associated factors among type 2 diabetes patients in Felege-Hiwot and Debre Markos Referral Hospitals using a longitudinal data analysis approach of a generalized linear mixed model. This study accounts for the glycemic control variation of patients over time and thus maximizes the amount of information drawn from the data.

## Results

### Characteristics of study participants

A total of 191 T2DM patients with 1740 observations were included in the analysis. There were more male patients (61.8%) than females (38.2%). The mean (SD) age of patients at the start of treatment was 57.9 (± 10.5) years. Two-thirds of the patients 144 (75.4) lived in urban areas. About 126 (65.9) and 122 (63.9) of the respondents had developed diabetes complications and hypertension, respectively (Table 1). The overall mean weight of T2DM patients was 73. 29 ± 5.53 Kg, with a minimum of 58 kg and a maximum of 87 kg (Table 1).

**Table 1:** Population characteristics for 191 type 2 DM patients with 1740 glycimic control stataus observations at Felege Hiwot and Debre Markos Referral Hospital, December 2014-January 2020.

Variable	Categories	Frequency (%)
Gender	Female	73(38.2)
	Male	118(61.8)
Residence	Rural	47(24.6)
	Urban	144(75.4)
Age in years	Mean (± SD)	57.89(± 10.47)
Weight in kg	Mean (± SD)	73.29(± 5.53)
DBP in mm Hg	Mean (± SD)	78.50(± 11.45)
SBP in mm Hg	Mean (± SD)	124.85(± 17.49)
FBS in mg/dl	Mean (± SD)	137.35(± 81.64)
Hypertension comorbidity	Yes	122(63.9)
	No	69(36.1)
Diabetic complication	Retinopathy	26(13.6)
	Nephropathy	38(19.9)
	Neuropathy	30(15.7)
	Stroke	8(4.2)
	Coronary artery disease	19(9.9)
	Prepral artery disease	5(2.6)
	No	65(34)

The overall mean duration of T2DM patients since diagnosis was 43.25 ± 9.57 months, with a minimum of 6 months and a maximum of 60 months (Table 2).

**Table 2:** Cross tabulation and Univariate analysis of glycemic control status for 191 patients with 1740 glycimic control status observations at DMRH and FHRH, December 2014–January 2020.

Variable	Catagories	Overall proportion of Glycemic control from total observation		p-value
		Poor (%)	Good (%)	
Gender	Female	259(35.8)	368(36.2)	0.833
	Male	465(64.2)	648(63.8)	
Residence	Rural	180(24.9)	143(14.0)	< 0.000
	Urban	544(75.1)	873(86.0)	
Age	38–50	176(24.3)	318(31.3)	0.001
	51–59	179(24.7)	237(23.3)	
	60–66	176(24.2)	231(22.8)	
	67–80	194(26.8)	230(22.6)	
Treatment	Insulin alone or insulin plus oral agents	123(17.0)	193(19.0)	0.522
	More than one oral agent	104(14.4)	161(15.9)	
	One oral agent	497(68.6)	661(65.1)	
Proteinurea	Negative	328(45.3)	576(56.7)	< 0.000
	Positive	396(54.7)	440(43.3)	
Weight	58–69	105(14.5)	380(37.4)	0.069
	70–73	139(19.1)	253(24.9)	
	74–77	219(30.2)	217(21.4)	
	78–87	262(36.2)	166(16.3)	
DBP	< 90	479(66.1)	778(76.6)	0.001
	≥ 90	245(33.9)	238(23.4)	
SBP	< 140	498(68.6)	827(81.4)	< 0.000
	≥ 140	226(31.2)	189(18.6)	
Creatinine, mg/dl	Mean (± SD)	1.26(± 0.28)	1.21 ± (0. 27)	< 0.000
Duration per visit, months	Mean (± SD)	1.5(± 1.7)	1.4(± 1.3)	0.002
Duration since diagnosis, months	Mean (± SD)	43.1(± 9.9)	43.4(± 9.3)	0.043

### Prevalence of glycemic control in T2DM Patients

**Figure 1:**
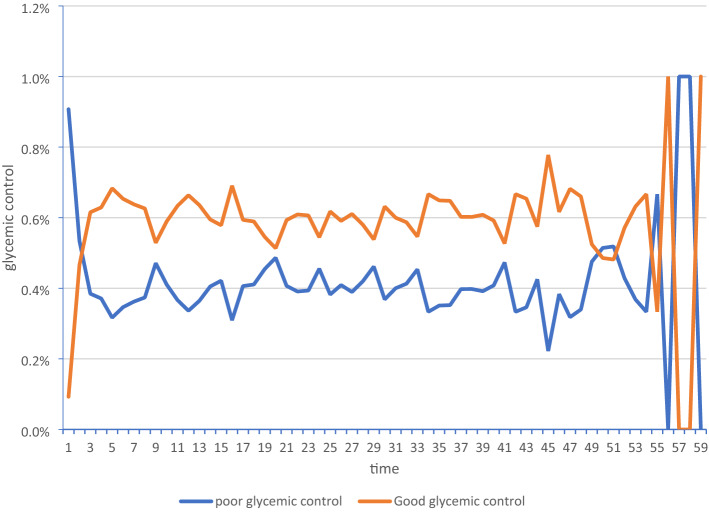
Fluctuation in glycemic control over time among type 2 diabetes patients at Felege Hiwot and Debre Markos Referral Hospital, December 2014–January 2020.

The overall prevalence of uncontrolled glycemic control was 41.6% (95% CI 40.3–42.9%) (Table 2). The overall mean of fasting blood sugar was 137.35mg/dl ± 81.64 (Table 1). The proportion of glycemic control fluctuated over time. Relatively speaking, the prevalence of well-glycemic control progressively rose with follow-up time (Fig. 1). Hence, 23.25% of the variation was explained in the GLMM model due to the incorporation between patient glycemic control variation over time (Table 3).

**Table 3:** Results of Generalized Linear Mixed model with logit link for factors associated with glycemic control for 191 type 2 diabetes mellitus patients with 1740 glycimic control status observations.

Variables		COR (95%CI)	Multivariable analysis	
			AOR (95%CI)	P-value
Residence	Rural	0.492(0.427, 0.567)	0.528(0.454, 0.614)	< 0.000
	Urban (ref)			
Age	≤ 50	1.521(1.303, 1.774)	1.500(1.267, 1.776)	< 0.000
	51–59	1.115(0.952, 1.306)	1.249(1.057, 1.476)	0.027
	60–66	1.111(0.947, 1.302)	1.187(1.004, 1.403)	0.030
	≥ 67 (ref)			
Proteinurea	Negative	1.580(1.412, 1.769)	1.368(1.211,1.546)	< 0.000
	Positive(ref)			
Weight	≤ 69(ref)			
	70–73	0.865(0.710, 1.054)	0.947(0.766, 1.171)	0.315
	74–77	0.794(0.677, 0.932)	0.898(0.757, 1.064 )	0.764
	≥ 78	0.543(0.457, 0.645)	0.729(0.603, 0.881)	0.015
DBP	< 90	0.678(1.484, 1.898)	1.295(1.101, 1.522)	0.001
	≥ 90 (ref)			
SBP	< 140	1.981(.740, 2.256)	1.601(1.352, 1.895)	< 0.000
	≥ 140 (ref)			
Creatinine	continuous	0.452(0.365, 0.561)	0.523(0.415, 0.660)	< 0.000
Duration per visit	Continous	0.943(0.908, 0.980)	0.949(0.913, 0.987)	0.002
Duration since diagnosis	Continous	1.004(0.998, 1.010)	0.992(0.985, 0.998)	0.042
Time	Continous	1.011(1.006, 1.016)	1.014(1.010, 1.019)	< 0.000
Cov Parm	Subject	Estimate	Standard Error	
AR (1)	ID	0.215	0.014	
Residual		0.997	0.020	

*AR (1)* autoregressive order one, *AOR* adjusted odd ratio, *COR* crud odd ratio, *CovParm* covariance parameter, *DBP* diastolic blood pressure, *SBP* systolic blood pressure, *ref* reference group, *95%CI* 95% Confidence interval, *P-value* Probability value.

### Factors associated with good glycemic control among T2DM patients

In bivariable analysis, the variable residence, age of the patient, weight, duration of T2DM since diagnosis, duration of T2DM per visit, follow-up time, proteinuria, diastolic blood pressure, systolic blood pressure, and serum creatinine were significantly associated with good glycemic control among T2DM patients at 0.2 level of significance (Table 2).

In multivariable analysis, residence, age, weight, duration of T2DM since diagnosis, duration per visit, follow-up time, proteinuria, diastolic blood pressure, systolic blood pressure, and serum creatinine had significant effects on good glycemic control at 0.05 level of significance (Table 3).

The odds of good glycemic control among patients who lived in rural areas were 47.2% lower compared to those patients who lived in urban areas (AOR 0.528, 95% CI 0.454, 0.614). The odds of good glycemic control for patients whose ages were 38–50, 51–59, and 60–66 years were about 50%, 24.9%, and 18.7% higher as compared with those patients whose ages were 67–80 years old, (AOR 1.500, 95% CI 1.267,1.776), (AOR 1.249, 95% CI 1.057, 1.476) and (AOR 1.187, 95% CI 1.004, 1.403), respectively.

The odds of good glycemic control for negative proteinuria T2DM patients were about 36.8% higher compared to positive proteinuria T2DM patients (AOR 1.368, 95% CI 1.211, 1.546). The odds of good glycemic control were lower by 47.7% when serum creatinine increased by one mg/dl (AOR 0.523, 95% CI 0.415, 0.660).

The odds of good glycemic control among patients whose diastolic blood pressure was < 90 mmHg were about 29.5% higher as compared with those patients whose diastolic blood pressure was ≥ 90 mmHg (AOR 1.295, 95% CI 1.101, 1.522). Similarly, the odds of good glycemic control for patients whose systolic blood pressure was < 140 mmHg were about 60.1% higher compared to those patients whose systolic blood pressure was ≥ 140 mmHg (AOR 1.601, 95% CI 1.352, 1.895).

The odds of good glycemic control for patients whose weights were 78–87 kg were about 27.1% lower compared to those patients whose weights were 58–69 kg (AOR 0.729, 95% CI 0.603, 0.881). The odds of good glycemic control were lower by 5.1% when the duration per visit of the patients increased by one month (AOR 0.949, 95% CI 0.913, 0.987). Likewise, the odds of good glycemic control were lower by 0.8% when the duration since the diagnosis of the patients increased by one month (AOR 0.992, 95% CI 0.985, 0.998).

## Discussion

In this study, a generalized linear mixed model with autoregressive order one analysis was used to identify the determinant factors that affected good glycemic control among T2DM patients in two of the major hospitals in North West Ethiopia.

The study revealed that 58.4% of the patients had good glycemic control of blood glucose. The proportion of good glycemic control was comparable to the results reported in the Dilchora Referral Hospital, Ambo, Brazil, Iran, and Jordan^5,12–15^. In other studies, approved in Adama Medical College Hospital (35.9%), Ethiopia (31.1%), Referral Hospitals of Amhara Region (44.7%), Riyadh (32.3%), Al-Hasa (32.1%), Jazan (26%), Oman (35%), United Arab Emirates (31%), Kuwait (21.2%) and Rawalpindi (24%), good glycemic control was inferior unlike the current study^6–8,16–21^. The observed difference between this and other studies could be because of the difference in sample size, study design (this study used longitudinal data, while other studies used cross-sectional data), and the operational definition used (this study uses fasting blood glucose levels to categorize glycemic control, while other studies use hemoglobin A1c to categorize glycemic control). Besides, the presence of great variation in socioeconomic, cultural, and lifestyle of the study populations across different studies may play a great role in the observed difference.

The odds of good glycemic control among patients who lived in rural areas were 47.2% lower compared to those patients who lived in urban areas. The finding is in line with prior research studies^22–24^ and contradicts with the study conducted in Ethiopia, which reported no significant association between residents with poor glycemic control^5–9^. A possible reason for this finding might be due to lower awareness of treatment adherence among persons living in rural areas^25^. Besides, the majority of patients who live in rural areas may have lower educated levels.

The odds of good glycemic control for patients whose ages were 38–50, 51–59, and 60–66 years were about 50%, 24.9%, and 18.7% higher as compared with those patients whose ages were 67–80 years old, respectively. This finding agrees with the findings from India and Ethiopia^8,26^ and contradicts the study conducted in Ethiopia, which reported no significant association between age with poor glycemic control^5–7^. The possible cause for this finding is because of the happening of diabetes-related complications within higher ages^27^. This implies that older age not only increases the hazard of chronic illness; the supervision of the illnesses also becomes difficult.

The odds of good glycemic control for negative proteinuria T2DM patients were about 36.8% higher compared to positive proteinuria T2DM patients. The justification for this study is that positive proteinuria was an increasing the risk of vascular complications^9^, which may lead to poor glycemic control^28,29^.

The odds of good glycemic control decreased by 47.7% when serum creatinine increased by one mg/dl. The reason for this study is that increasing serum creatinine leads to an increase in the risk of vascular complications^9^, which leads to poor glycemic control^28,29^.

Patients whose diastolic blood pressure was < 90 mmHg had higher odds of good glycemic control compared to those patients whose diastolic blood pressure was ≥ 90 mmHg. Similarly, patients whose systolic blood pressure was < 140 mmHg had higher odds of good glycemic control compared with those patients whose systolic blood pressure was ≥ 140 mmHg. This result agrees with previous findings^8,9,30,31^ and contradicts the study conducted in Ethiopia, which reported no significant association between blood pressure control with poor glycemic control^7^. The possible justification for this study is due to the additional antihypertensive pill burden and the complication inhibiting the utilization of peripheral glucose, which finally increases the fasting blood glucose level^32^.

The odds of good glycemic control were lower by 5.1% when the duration per visit of the patients increased by one month. Likewise, the odds of good glycemic control were lower by 0.8% when the duration since the diagnosis of the patients increased by 1  month. This study is in line with the findings of the previous study^8^ and contradicts the study conducted in Ethiopia, which reported no significant association between duration since diagnosis in patients with poor glycemic control^5–7^. The possible justification for this study is that progressive impairment of insulin secretion through time because β cell failure could lead to poor glycemic control.

Patients whose weights were 78–87 kg had lower odds of good glycemic control compared to those patients whose weights were 58–69 kg. This finding was supported by previous studies^6–8,30^ and contradicts the study conducted in Ethiopia, which reported no significant association between the weight of patients with poor glycemic control^5,9^. The possible justification is that higher weight will increase blood sugar and increase the risk of diabetes complications.

The main limitation of the study is the limited information on predictors: such as family history, medication adherence, patients’ perception of health, the type of intervention, including the type of physical exercise, and nutritional status of a patient that may have influenced the outcome variables. Due to a lack of data on these predictors, we have excluded them from the analyses. Therefore, more public health and epidemiology research is needed to examine the impact of these variables to identify new risk factors of glycemic control for T2DM patients by using the HbA1c test, which is a good predictor of glycemic control over a long time.

## Conclusions and recommendations

This study revealed that the overall prevalence of poor glycemic control was high at Debre Markos and Felege Hiwot Referral Hospital. Living in a rural area, older age (≥ 67 years), positive proteinuria, higher weight (≥ 78kg), higher serum creatinine levels, higher duration per visit, higher time duration of T2DM since diagnosis, and developing hypertension (SBP ≥ 140, DBP ≥ 90) were the predictors of lower good glycemic control achievements of T2DM patients. Therefore, strengthening and disseminating health education programs for diabetes patients at each follow-up visit on the importance of achieving optimal body weight, negative proteinuria, and controlling blood pressure to prevent and mitigate the complications resulting from poor glycemic control. Health professionals working in the hospital should provide good patient advice for type 2 DM patients: living in the rural area, older age (≥ 67 years), with higher serum creatinine levels, higher duration per visit and higher time duration of T2DM since diagnosis to maximize efforts on the prevention of T2DM complications and risk minimization resulting from poor glycemic control.

## Methods

### Study design, study area, and study period

An institutional-based retrospective follow-up study design was conducted at Felege-Hiwot and Debre Markos Referral Hospital with type 2 diabetes mellitus patients who were enrolled from December 2014 to December 2015. We have reviewed the chart of these patients until January 2020.

Felege-Hiwot Referral Hospital (FHRH) is found in Bahir Dar, the capital city of the Amhara Regional State, a region in the Northwest of Ethiopia, whereas Debre Markos Referral Hospital (DMRH) is found in Debre Markos, the capital city of East Gojjam zone, a region in the Northwest of Ethiopia.

These hospitals were selected because they are the only referral hospitals under the thematic research area of the finder (Debre Markous University). Besides, we expected that the chance of getting more recorded valid information was better than other non-referral hospitals because of the presence of experienced staff and modern laboratory equipment. As a result of this, we have selected those hospitals over others, purposively. In addition, these hospitals were geographically 264 km away. Thus, it is more likely to get an adequate size of type 2 diabetic patients from different socio-cultural and environmental conditions.

### Source and study population

The source population was all T2DM patients who were found at Felege-Hiwot and Debre Markos Referral Hospitals, whereas the study population was all type 2 diabetic patients aged 18 years or older who came to the hospital for diagnosis and follow-up from December 2014 to December 2015. We have reviewed the chart of these patients until January 2020. A total of 191 patients, who fulfilled the inclusion criteria of the study, with 5220 observations of FBS repeatedly measured values were changed in to 1740 glycemic control status observations. Since, we have used each of the three consecutives measured FBS to determine the glycemic control status of the patients.

### Inclusion and exclusion criteria

**Figure 2:**
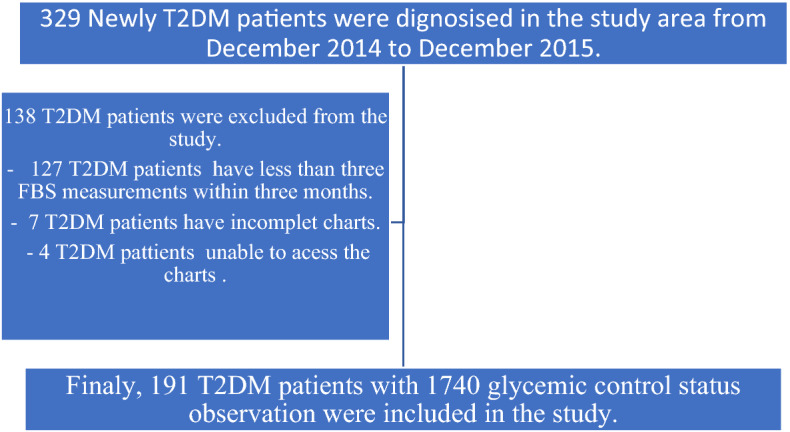
Summary of study participants recruiting flow chart.

T2DM patients with three fasting blood glucose measurements within three months and above the age of 18 years were included in the study, whereas the patient chart would not be available during the data collection period and patients with missing key predictor variables were excluded from the study. Finally, 191 T2DM patients met the inclusive critter of the study, whereas 138 T2DM patients were excluded from the study (Fig. 2).

### Study variables

The dependent variable was glycemic control status. The independent variables were socio-demographic variables (age, sex, residence), clinical characteristics (weight, duration of DM since diagnosis, duration per visit, specific type of drug regimen, comorbidity, DM complications), and physiological characteristics (creatinine, Systolic Blood Pressure (SBP), Diastolic Blood Pressure (DBP), and proteinuria).

### Operational definitions

Fasting Blood Sugar is blood glucose measured from venous blood after at least 8 hours of overnight fasting^33^.

*Good glycemic control* was defined as patients whose average fasting blood glucose measurement of three consecutive visits was between 70 and 130 mg/dL^33,34^.

*Poor glycemic control* was defined as patients whose average blood glucose measurement for three consecutive visits was above 130 or below 70 mg/dL^33,34^.

Protein urea is defined as positive if the urine albumin concentration is between 30 mg/24 h and 300 mg/24 h and negative if it is < 300 mg/ 24 h^9^.

### Data collection procedures and data quality control

Data were extracted by reviewing patient charts using a checklist. The data were collected by two nurses who had experience in diabetic follow-up and the required laboratory values were taken from the patient’s medical record. To control data quality, training was given to the data collectors and their supervisors. The data extraction checklist was pretested for consistency of understanding of the review tools and completeness of data items. The necessary adjustments were made to the final data extraction format. Finally, the filled formats were checked daily by the supervisor.

### Ethics approval and consent to participate

Ethical approval was obtained from the Natural and Computational Science College Research Ethics Committee (Debre Markos University), and permission was obtained from the medical directors of the Hospital. We confirm that all methods were carried out by relevant guidelines and regulations. Due to the retrospective nature of the study, the need for informed consent was waived by the Research Ethics Committee of Natural and Computational Science College (Debre Markous University), but the data were anonymous and kept confidential.

### Data analysis

Descriptive statistics were used to describe the percentage and frequency of patients with all covariates. In addition, a line graph was used to see the progression of glycemic control over time. A generalized linear mixed model with autoregressive order one covariance structure was used to identify the predictors of glycemic control among type 2 diabetic patients because AR(1) covariance structure has the best covariance structure than others in the current study. Since, it has the smallest AIC and BIC than other covariance structures (Table 4). The odds ratio was used to assess the association between glycemic control and risk factors. The 95%CI that did not include one or a P-value less than 5% was taken to identify a significant association between glycemic control and risk factors.

**Table 4:** Summarized value of Information criteria for 191 type 2 diabetes mellitus patients with 1740 glycimic control status observations at DMRH and FHRH, December 2014–December 2020.

Covariance structure	AIC	BIC	Generalized Chi-Square	Gener. Chi-Square / DF
Constant variance	22,726.33	22,726.53	5253.29	1.00
Compound symmetry	22,877.57	22,877.97	5299.18	1.01
Autoregressive order one	22,458.78	22,459.18	5251.35	1.00
Toeplitz	22,459.18	22,460.28	5252.52	1.00
Unstructured	22,890.72	22,891.32	5301.35	1.00

### Generalized linear mixed model

We used the extension of multiple binary logistics regression to include both fixed and random effects (subjects) in the generalized linear mixed model because the dependent variable had dichotomous natures as well as we have cheeked that there was no dispersion parameter in the data. GLMMs are generally defined such that, conditioned on the random effect $\upsilon$, the dependent variable $Y (\mathrm{glycemic control for the }{\mathrm{i}}^{\mathrm{th}}\mathrm{ type }2\mathrm{ diabetes }{\mathrm{patient}}^{\mathrm{^{\prime}}}\mathrm{s}),$ is distributed to a binomial distribution with its expectation related to the linear predictor $X\beta +Zu$ via a logit link function $g$:

$$g\left( {E\left| y \right|u} \right) = X\beta + Zu$$

Here ***X*** and β are the fixed effects design matrix, and fixed effects; ***Z*** and ***u*** are the random effects design matrix and random effects. Generalized linear mixed models enabled us to see heterogeneity between patients.

### Parameter estimation for generalized linear mixed models

The marginal quasi-likelihood method was used to estimate the model parameters. The complete likelihood for all observed data is formulated as^35^

$$\ln \,p(y,u) = \ln \int {p\left( {y|u} \right)} \;p\left( u \right)du$$

The likelihood function has no general closed-form, and integrating over random effects is usually extremely computationally intensive. In addition to numerically approximating this, integral, methods motivated by Laplace approximation have been proposed^36^.

To build the generalized linear mixed model analysis, using the procedure we followed, first, we did a bivariable analysis for each of the explanatory variables and based on statistical significance at 0.2 level of significance, the identified variables to be candidates for the multivariable analysis^37^. As naturally different identified factors/variables do not operate separately, multivariable analysis helps to control for confounders and analyze the effects of a factor in the presence of other factors in the model.

We used Akaike and Bayesian information criteria to select the appropriate generalized linear mixed model with its best covariance structure, and the model with the smallest AIC or BIC was considered the best fit^38,39^.

## Data Availability

The data sets analyzed in this study are available from the corresponding author on reasonable request.

## Abbreviations

ADA: America Diabetes Association
AIC: Akaike information criteria
AOR: Adjusted odds ratio
BIC: Bayesian information criteria
CHD: Coronary heart disease
CI: Confidence interval
COR: Crude odds ratio
DBP: Diastolic Blood pressure
DM: Diabetes Mellitus
DMRH: Debre Markos Referral Hospital
FHRH: Felege-Hiwot Referral Hospital
GLMM: Generalized linear mixed model
IDF: International Diabetes Federation
PAD: Peripheral arterial disease
SBP: Systolic Blood pressure
T2DM: Type 2 Diabetes Mellitus
